# Neglected Dislocation in Adults: A New Therapeutic Strategy for an Uncommon Condition

**DOI:** 10.3390/geriatrics8060117

**Published:** 2023-11-30

**Authors:** Juan Carlos García de la Blanca, Javier Cuarental García, Gonzalo Luengo, Rafael Martí, Luis Rafael Ramos

**Affiliations:** Orthopedic Surgery Department, Hospital Universitario 12 de Octubre, 28041 Madrid, Spain; javi_cuarental@hotmail.com (J.C.G.); gluenal@gmail.com (G.L.); marticiruelos@yahoo.es (R.M.); luisramospascua@gmail.com (L.R.R.)

**Keywords:** dislocation, neglected, hip, arthrodiastasis, Ilizarov

## Abstract

(1) Background: Neglected hip dislocation is an uncommon condition, especially in developed countries because dislocations are considered trauma emergencies and thus are treated early. They are usually treated with methods used in commonly occurring dislocations. The aim of this study is to detail a two-stage strategy for neglected hip dislocations in adults applied in a complicated case. (2) Case presentation: We present a complicated case of neglected hip dislocation in a patient with an associated neurological condition. Two-stage open reduction was performed combined with arthrodiastasis and Ilizarov external fixators. After complications requiring a third intervention, the joint was stabilized, and the patient has presented no more episodes to date. (3) Conclusions: Arthrodiastasis has been used to treat these conditions. Comparing it with the other methods described in the literature, it seems to be a good therapeutic strategy, especially in elderly patients. Because of the limited number of studies, we cannot establish the most efficient therapeutic method, but we believe that the described strategy can be useful for patients with this condition.

## 1. Introduction

Hip dislocations in adults are uncommon, and their occurrence is usually related to high-energy trauma, as atraumatic dislocations are very unusual and are associated with previous anomalies [1,2,3]. Because hip dislocations are considered trauma emergencies, they are usually treated with early reduction, improving the patient prognosis. Neglected dislocations are thus considered very rare conditions in developed countries with easily accessible healthcare [2,4].

Due to the low frequency of cases in clinical practice, there is no consensus on the therapeutic approach to neglected dislocations in adults, as the literature only reports isolated cases. Their management is based on that of traumatic, congenital or pediatric hip dislocations, which have been more commonly reported and extensively described [2,5,6,7].

The natural time progression of dislocations may involve the appearance or aggravation of musculoskeletal deformities in nearby joints; therefore, it is important to find the optimal therapeutic alternative that improves patient prognosis and reduces recurrence and associated complications [5].

The aim of this study was to describe the evaluation and treatment method used in a complicated case of neglected hip dislocation, encompassing the entire therapeutic arsenal available to the orthopedic surgeon, and to review the existing literature.

## 2. Case Presentation

A 69-year-old woman was referred to orthopedic surgery with a clinical picture of fixed right hip flexion and adduction and no obvious trauma history. The patient was undergoing rehabilitation of a contralateral ankle fracture, immobilized 9 months before, the time at which the beginning of progressive joint deformity was attributed.

The patient had a neurological disease history, i.e., akinetic-rigid syndrome (2 years of evolution). In addition, she had undergone surgery 3 years prior for myelopathy secondary to cervical disc herniations, with triple discectomy and C4–C7 anterior fusion.

Before suffering from the progressive neurological condition, she was independent in daily activities, with no other relevant history. However, her quality of life was severely affected by stiffness, gait disorder, and right hip deformity, which forced her to spend most of her time in bed; therefore, ambulation could not be assessed.

**Figure 1:**
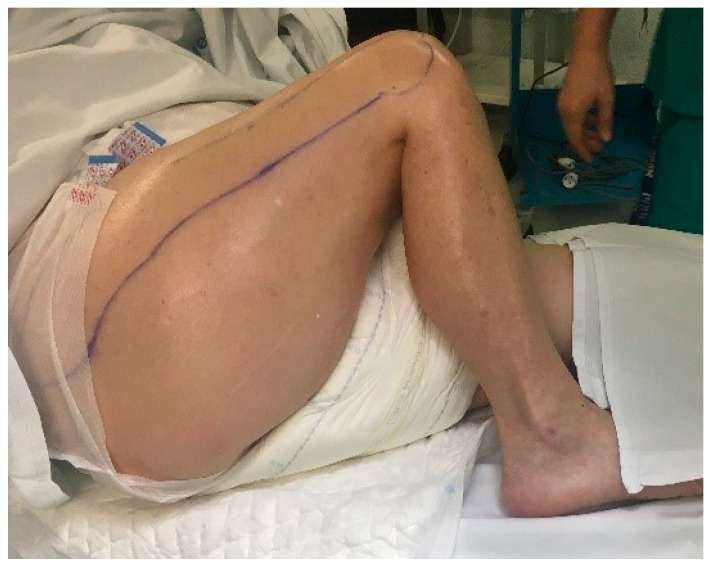
Patient at the time of diagnosis. She had fixed 90° hip flexion and fixed 100° knee flexion.

The initial diagnosis was obtained via physical examination, observing the fixed 90° right hip flexion with adduction and internal rotation and the fixed 100° right knee flexion (Figure 1). There was a loss of active mobility in both joints, while that of the ankle was preserved. There was a tendency to joint stiffness in the contralateral leg; although, it retained mobility, except for an equinus deformity of the left ankle.

**Figure 2:**
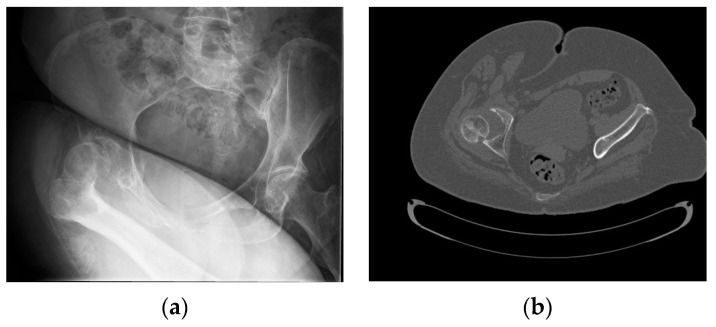

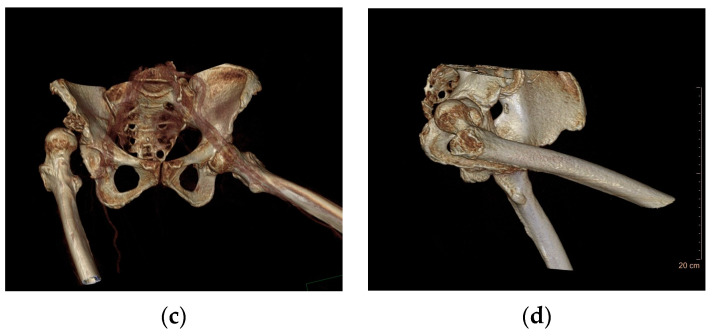
(**a**) Initial pelvic radiograph in which posterosuperior hip subluxation is observed without fractures; (**b**) Computerized Tomography (CT) scan (transverse). There is no presence of avascular necrosis of the right hip. (**c**,**d**) 3D reconstruction from the CT scan.

**Figure 3:**
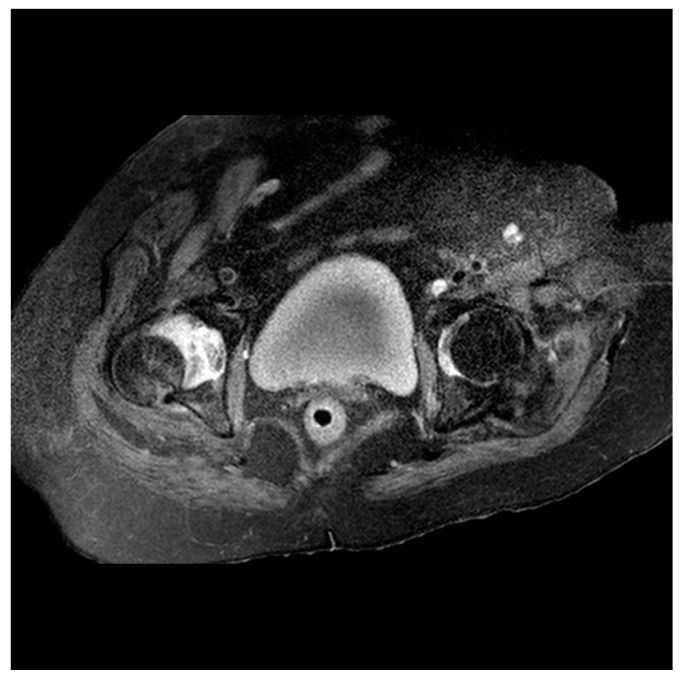
Pelvic Magnetic Resonance Imaging (MRI). The viability of the femoral head and the absence of fractures are confirmed.

The initial radiograph showed a practically total right coxofemoral subluxation, with no associated fractures (Figure 2a). Computerized Tomography (CT) and Magnetic Resonance Imaging (MRI) were requested to rule out avascular necrosis and to observe in greater detail the relative positioning of the femur and pelvis and the absence of fractures (Figure 2b–d and Figure 3).

### 2.1. Two-Stage Open Reduction

In the first surgical stage, two external fixation frames (TrueLok, Orthofix Medical Inc., Lewisville, TX, USA) were applied to extend the hip and knee progressively.

**Figure 4:**
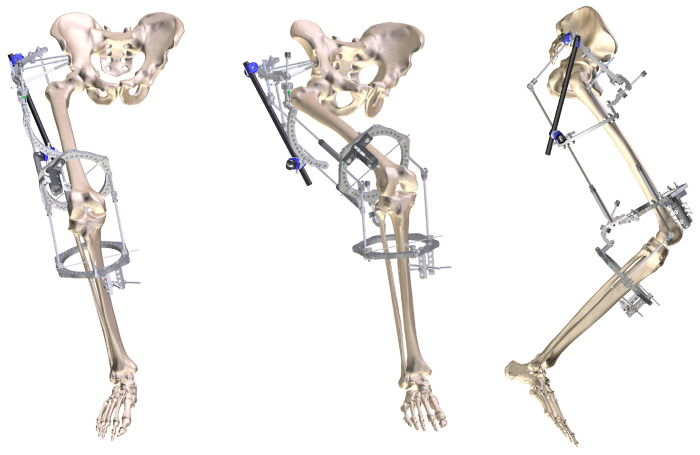
Frontal and lateral 3D reconstruction of the two-plane external fixation assembly used in the first surgical stage (TrueLok Hex, Orthofix).

The hip assembly consisted of two half rings: the proximal attached to the pelvis above the acetabulum and the distal attached to the femoral shaft (Figure 4). This arrangement allowed relative movement of the two articular ends. To control the assembly, the rings were connected through a system of hinges and distractors allowing progressive joint diastasis and extension. A similar system was applied to the knee, but with two full rings attached to the femur and proximal third of the tibia. These were connected by two hinges aligned with the knee flexion axis and a rear motor to apply the extension force. A bone fixation was performed with hydroxyapatite-coated screws and reinforced with femoral and tibial metaphyseal wires.

To reduce the resistance exerted by soft tissues and facilitate joint distraction, an adductor tenotomy was performed after applying the external fixation frames.

The prescribed postoperative distraction was 1 mm daily at the hip and 1 degree daily at the knee, achieving a progressive hip reduction and a gradual improvement in the ipsilateral knee extension.

In postoperative recovery, superficial infections were detected both in the trajectory of the pelvic screws and the tenotomy incision and were resolved after treatment with specific oral antibiotic therapy.

Two months after the first surgery, the second surgical stage was performed, consisting of an open hip reduction. After removing the external pelvic fixator, a lateral Hardinge approach was used, including myotomy of the iliopsoas and anterior rectum femoris. The femoral head had an acceptable macroscopic appearance both in morphology and blood supply.

After joint reduction, an abduction splint was maintained for 2 weeks. The knee fixator remained in position for 3 months, obtaining a passive mobility of 0–90° under anesthesia.

### 2.2. Complications after Treatment

During rehabilitation, the patient suffered a spontaneous anterior dislocation, not amenable to a closed reduction. When analyzed using CT, a deep infection involving the gluteal and adductor muscles was discovered, leading to a third surgery with the washing of the lesion and an open reduction following the same approach described earlier. Aggressive antibiotic therapy was administered to eradicate the infection.

Two weeks later, another dislocation occurred. In a new intervention, spontaneous reduction of the joint was observed, remaining stable in flexion, rotation, and abduction. The hip was immobilized with an abduction splint with the foot in internal rotation for 1 week, replacing it later with an abduction orthosis.

### 2.3. Case Follow-Up

Four months after the first surgery, the hip remained reduced, but there was a tendency to adduction and external rotation despite the orthosis. The patient tolerated sitting but attempts to initiate standing and walking were unsuccessful due to the progression of her neurological disease. She was diagnosed with a hyperintense spinal cord lesion (T8–T10) consistent with transverse dorsal myelitis, which was not treated surgically and caused spastic paraparesis in the patient. In the left lower extremity, irreducible equinus persisted (40° dorsal flexion and 70° plantar flexion), with mobility maintained in the hip and knee, with 4/5 proximal muscle strength and 5/5 distal muscle strength according to the scale proposed by the Medical Research Council.

**Figure 5:**
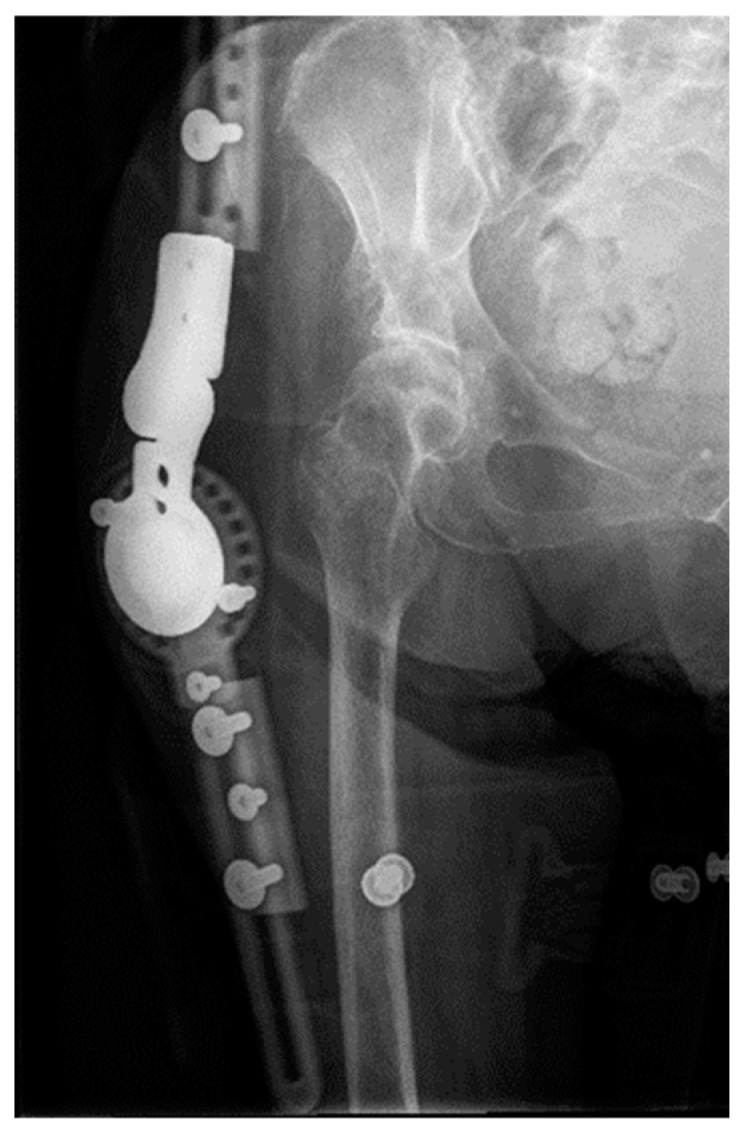
Control radiography at 20 months after the first intervention.

Currently, 20 months after the first intervention, the hip is still reduced without new episodes of dislocation, showing adequate joint congruency and the preservation of morphology and blood supply to the femoral head (Figure 5). Because of the high risk of dislocation associated with her neurological condition, the patient is maintained with an abduction orthosis.

Despite the therapeutic success achieved with the two-stage reduction, her current functional situation is poor due to her neurological condition, still of unknown origin and with an unknown prognosis.

## 3. Discussion

The hip joint is intrinsically stable because of its congruency and the soft tissues surrounding it. Therefore, hip dislocation is rare and usually associated with high-energy trauma. However, some biological factors predispose individuals to dislocations, such as an increase in the cervical–diaphyseal angle or a decrease in femoral anteversion (which could become retroversion in extreme cases) [2,3,8]. Likewise, patients with neurological disorders commonly experience hip dislocations [9].

Hip dislocations must be considered clinical emergencies due to the importance of time in their progression. The final prognosis is better when treated in the first 6 h after the originating event, ensuring the viability of the femoral head and reducing the risks of avascular necrosis and secondary osteoarthritis [5,10,11,12,13].

With our patient, various neurological and psychosocial conditions caused a months-long delay in the hip dislocation diagnosis. However, the delay did not result in osteonecrosis of the femoral head, perhaps due to its gradual progression.

Therapeutic strategies have traditionally followed those applied in commonly occurring hip dislocations. Specifically, although also considered rare conditions, hip dislocations in pediatric patients represent the greatest source of bibliographic information. Pediatric hip dislocations differ from those of adults because they have a better prognosis and are generally easily reducible.

There seems to be a consensus on the approach to attempting closed reduction first when reaching an early diagnosis [14]. Some authors advocate using trans-skeletal traction to reposition the femoral head in the acetabulum, which has been used in some adult patients, achieving satisfactory results when combined with closed reduction maneuvers [15]. However, extending its use to pediatric patients is not suggested. The efficacy rates are below 50%, and its benefits do not exceed the complications, including long hospital stays, difficult postoperative care, and complex joint repositioning due to pelvic instability [6,10].

In cases with soft tissue damage—additional injuries in the ipsilateral lower extremity, persistent instability, or prolonged dislocation over a long period—it is recommended to avoid closed reduction and choose open reduction. This allows the release of soft tissue and the removal of fibrous tissue from within the acetabulum; this approach may also be accompanied by joint repositioning methods [5,7,16].

Total hip arthroplasty has been described to treat hip dislocations in adults, both posterior neglected dislocations—analogous to our case—and congenital dislocations prolonged over time, in which case the approach is accompanied by an osteotomy or external fixation to complement the reduction [17,18,19]. Although the overall results are favorable, showing improvement in gait, pain, and mobility, the high mechanical stress to which the prosthetic implant is subjected cannot be underestimated, which can lead to subsequent loosening and surgical revisions. Especially in cases with such an extreme knee fixed flexion contracture as presented by our patient, not even the use of implants that guarantee a higher theoretical joint stability, such as bimodular femoral stems, or those recommended for neuromuscular pathologies or a high risk of dislocation, such as dual mobility cups, would be advisable [7,8,11,16,20,21].

The Girdlestone technique, first described to treat hip joint sepsis, has been used to treat neglected dislocations, accompanied by skeletal traction. Treatment success in the two cases considered may be associated with the patient’s age because the approach is well tolerated in young people, in addition to allowing future prosthetic replacement with arthroplasty [22,23]. However, the technique involves limb shortening; therefore, this strategy is not appropriate in elderly patients.

As an alternative method, reconstructive osteotomies of both the pelvis and femur can be used. Some authors have obtained satisfactory results using external fixation to combine abduction–extension osteotomy of the proximal femur with distal varisation–extension osteotomy [8].

Our method of choice, arthrodiastasis, has been used since 1979 to perform joint distraction by external fixation with monolateral or Ilizarov-type devices to treat multiple conditions, including femoral neck fractures, osteoarthritis, sequelae of Perthes disease, epiphysiolysis, or chondrolysis [6,8,24,25,26,27].

Using fixators for distraction reduces patient traction discomfort, allows early hip mobility, and facilitates subsequent care, in addition to providing positive effects on bone regeneration and chondroprotection both on the femoral head and acetabulum and increasing the blood supply to adjacent tissues. Moreover, it can ease the challenge faced by the patient’s caretaker in providing assistance, which can be rather difficult in cases like the one presented here [6,25,26].

Our therapeutic plan, based on the approach established by two congenital hip dislocation studies [6,28], consisted of achieving joint reduction primarily via arthrodiastasis with a circular external fixator, for which a custom-made assembly was planned and prepared according to the characteristics of the patient. Once the femoral head was placed in its normal position in the acetabulum, an open reduction was performed in a second surgical stage.

One of the main complications of the treatment of neglected dislocations is the retraction of the soft tissue and fibrous tissue occupying the cavity of the acetabulum, sometimes making closed reduction impossible. The release of the soft tissue is especially important in patients with spasticity; therefore, in our case, we included surgical gestures such as tenotomies of the iliopsoas and anterior rectum femoris [6,28,29].

The design of the fixation devices was customized according to the characteristics of the patient, securing the structures via several levels of fixation to achieve greater stability. To avoid applying a high-intensity protocol, the speed of joint distraction was set at 1 mm daily for 60 days, until joint repositioning was achieved [6,24,26,28]. In this way, progressive traction was applied, inducing tissue neovascularization and avoiding sudden gestures that could cause added pain and stiffness [30].

The main complication of arthrodiastasis described in the literature is the infection of the screw trajectories, with an incidence between 21% and 95% [24,26,28]. Our patient suffered an infection attributed to the fixation points, subsiding at first but later worsening and involving the soft tissue. Despite this and the two redislocations that occurred during recovery, joint stability was finally achieved, with no other notable incident 20 months after the start of treatment.

Given the neurological condition of our patient, we were unable to implement a conventional physical rehabilitation strategy. The development of systems that simulate gait conditions is of significant medical interest, as it allows patients who cannot walk autonomously to benefit from their physiological advantages. Ensuring that these systems are accessible in all healthcare centers is crucial to tailor care to the specific and complex needs of these patients [31].

Due to the small number of cases published, there is insufficient evidence to determine the most effective method for treating neglected hip dislocations in adults. Orthopedic surgeons must consider the time of progression of the lesion and patient conditions when determining the appropriate strategy.

## 4. Conclusions

We presented a case of neglected hip dislocation treated using arthrodiastasis with a circular fixator for joint preservation associated with soft tissue release techniques and open reduction in a second surgical stage. The patient suffered recurrence, finally stabilized, and remains stable.

## Data Availability

Data is unavailable due to privacy restrictions.
